# Syphilis of the Mouth

**Published:** 1905-03

**Authors:** Henry C. Boenning

**Affiliations:** Philadelphia


### SYPHILIS OF THE MOUTH.[1]

BY HENRY C. BOENNING, M.D., PHILADELPHIA.

Syphilis of the mouth is of very common occurrence, and of especial interest to the dentist because of his exposure to infection.

The lesions are of the most protean character; some are highly contagious; hence a discussion of the features of this disease is of the greatest practical advantage to the members of your profession.

The question, "What is Syphilis?" has been asked from time immemorial. Syphilographers contend that much of David's physical distress and Job's diseased body was due to syphilis; and that the classics testify to the existence of this disease in the remotest eras of civilization.

---

[1] Read before the Chester and Delaware County Dental Society, October 26, 1904.

Certain it is that syphilis is to-day the most commonly met and most widely distributed disease. It is no respecter of persons, or of rank in society. The rich and high-born share equally with the poor and plebeian the susceptibility to infection.

The cause of syphilis is a germ. This has been established beyond controversy; but whether that germ is an animal or a vegetable micro-organism has not been shown.

From time to time, up to the present, the germ of syphilis has been "announced," only to find that it fails to meet Koch's law, but eventually it will be isolated, and a new treatment, by probably an antisyphilitic toxin, will be inaugurated, to the enormous benefit and relief of the syphilitic, whose afflictions are often greater than those of the leper.

Lustgarten's bacillus, supposed by many to be the cause of syphilis, failed in the laboratory to prove its claims; but with each exclusion we gain a better understanding of the action and probable identity of the germ responsible for this malady.

Syphilis is a disease of different stages. This conforms with nearly every zymotic disease in which the micro-organism has been satisfactorily demonstrated.

But the stages of syphilis extend over periods of months and years; whereas those of most diseases are limited to days and weeks. Thus, in typhoid we have the period of incubation of the germ, the prodromal period, the stage of invasion, the period of intestinal lesions, and the active disease, terminated at the end of three weeks, when begins the tardy and precarious stage of recovery by lysis. In yellow fever we have the stage of invasion, often fulminant, the period of lull or abatement, often deluding the patient into the conviction that recovery has set in, only to be followed in many cases by the dreaded black vomit and death—all phases of the disease terminated in a week or ten days.

In syphilis the stages and periods are as strongly marked but more greatly extended, and are viewed over a long lapse of time. They are months apart, years apart, and often extend throughout life.

Syphilis of the mouth may be of any stage,—primary, secondary, or tertiary; and not infrequently we meet here also the lesions of hereditary syphilis.

Primary syphilis is chancre and bubo. The initial point of infection is the chancre; and about the mouth it may occur on the lips (usually the lower), the gums, the tongue, and the tonsil. Other parts of the oral cavity, however, may be infected.

The mode of infection is direct and mediate.

Direct infection means infection-contact of the primary lesion, or contagious secondary lesion, or infected blood during either of these stages. Infection is greatly facilitated if the recipient has an abrasion of the part infected; in truth, it is held by many that a surface of unbroken continuity is invulnerable to infection, but this is fallacious. A subject with abundant mucous patches of the oral cavity can convey infection by a kiss. Any number of cases of specific infection are recorded in which the subject has been infected by some vessel or agent used by the syphilitic. This is mediate infection.

One of the most extraordinary cases of chancre about the oral cavity I ever saw was the case of a gentleman of unquestionable moral integrity. The source of infection was a mystery. The chancre involved the gum near the lower right lateral incisor. After a week of the most patient inquiry, he came to my office and told me that he had noticed the day previously that the clerk at the desk of the restaurant which he patronized picked a toothpick from the tray on the desk, and, after using it, tossed it back. He saw him do this several times. I instructed him to notify the manager, and, together they watched the cashier, and saw him use toothpick after toothpick, tossing some to the floor and some back upon the tray. The young man was notified to stop the habit he had acquired, and the toothpicks were removed from his desk, and at the request of my patient he was brought to me for an examination. His mouth was everywhere covered with mucous patches, and a general examination showed a virulent case of syphilis of about three months' standing. A contaminated toothpick was, in this case, undoubtedly the mediate means of infection.

Primary infection, the chancre, is then the point at which the syphilitic poison is introduced. This excepts, of course, congenital syphilis.

As has been said before, the donor of the disease may infect the non-syphilitic by the virus from a chancre, by the virus from a mucous patch, by discharges from secondary lesions (the so-called moist, secondary manifestations), by the blood during the primary and secondary stages, but not by the normal secretions, such as the saliva or those of the skin.

The chancre may appear in one of several different forms. Of these the deep, or conical, or Hunterian chancre, the chancrous erosion, and the dry chancre, or infecting papule, are the commonest. They all have one thing in common, and that is in their development they cause such irritation that marked induration takes places about and beneath the base of the chancre.

The chancrous erosion and the Hunterian chancre are those met with about the oral cavity. Very soon after the appearance of the chancre enlargement takes place in the lymphatic glands directly associated with the base of the sore. This is due to a lymphoid adenitis, and to this is due the bubo, or specific lymphatic tumor associated with chancre as primary syphilis.

An ulcer within the mouth or on the lip, of a duration of some weeks, and associated with a tumor of the lymphatic glands in the vicinity, should give rise to a suspicion of the presence of primary syphilis, especially if the person be a young man or woman, and the patient should be carefully examined and interrogated as to the cause of the ulcer.

The induration in chancre, especially of the lip, is peculiar. If it be the deep, or Hunterian type, and you take the parts between your fingers, it feels as if you held a bullet, in the top of which, extending toward the centre, is the ulcerating chancre.

In the chancrous erosion the induration is often referred to as parchment induration—it imparts the feel of a thickened layer. It is superficial, but of considerable area.

The victim of syphilis, after about six weeks of primary syphilis, begins to show some evidences of constitutional infection. He complains of slight fever and a general indisposition. If seen at this time the chancre has probably cicatrized, the bubo diminished in size, but the lymphatic glands at large have become involved, and a general lymphatic adenitis results. The lymphatics can often be felt as thickened cords. Sometimes when the lymphangitis is more marked they can be seen as red lines over various parts of the body. The lymphatic glands above the internal epicondyle and about the neck—especially behind the sterno-mastoid—and the post auricular glands are almost invariably involved, and form valuable diagnostic signs. They feel like small pebbles beneath the skin.

Some morning the patient observes, after exertion, perhaps, or after a bath, that his skin is mottled, and on closer examination he finds himself spotted like a leopard; but the spots are rose-colored, and disappear temporarily under pressure. This is the earliest syphiloderm, and is usually regarded as ushering in the secondary stage of the disease.

In rapid sequence we have the papular eruption, presenting a strongly marked eruption across the forehead, and known popularly as the "corona veneris." All these syphilodermata have a peculiar dull copper-color, as a rule.

Then the pustular and tubercular syphilides follow, often in rapid succession, sometimes so rapid that the next syphilide appears before the former disappear, and this gives rise to the remarkable appearance of a variety of eruptions *co-existent*, and known as the polymorphous syphiloderm.

Even before the skin manifestations occur there is present pain in the bones, most severe at night.

During this stage we also have the secondary iritis, alopecia, local and diffuse analgesia, and, most important to you, the throat and mouth complications.

Syphilitic sore throat is often an early secondary symptom, and is generally associated with mucous patches of the tongue, pillars of the pharynx, uvula, and inside of cheeks.

These oral patches are virulently contagious. Many dentists have become infected from them,—have developed chancre of the finger, and have gone through all the stages of this disease.

What is the mucous patch? It is a syphilitic rash in those positions where there is heat and moisture—where the epithelium is macerated. Hence it is found about the natural orifices; also beneath the breasts in women; also within the mouth and vagina.

Remember that the secondary moist manifestations are virulently contagious, and that if you put your finger into the mouth of a patient having mucous patches, and you have an abrasion on your finger, you are almost certainly sure to become infected, unless there be some special or exceptional immunity.

Mucous patches within the mouth are grayish-looking, surfaces slightly raised, and showing an abundance of macerated epithelium. They are seldom symmetrically disposed, their irregularity of distribution being a diagnostic sign. A little later they ulcerate, the ulcers frequently existing as fissures. About the uvula they often run a rapid course, and destruction of portions of the soft palate is frequently the result. Deep ulceration of the pharynx is very common. The roof of the mouth is often involved, generally among the later secondary lesions, and frequently coexisting with gummatous nodules of the hard palate. Unless now the most active treatment is employed, extensive destruction of the hard palate may ensue.

Tertiary syphilis may come on as early as the second year of this disease; sometimes it is many years later before its lesions develop.

The tertiary lesions are not generally contagious, and when they develop about the oral cavity are of the gummatous and tubercular varieties. These often lead to extensive necrosis of the hard palate; less frequently of the lower jaw. The bones of the nose are most vulnerable, and the disease often invades the air sinuses at the base of the skull. The tongue and salivary glands occasionally become affected with tubercular syphilis, a condition simulating hard cancer and sometimes mistaken for it.

Hereditary syphilis is, unfortunately, often seen. It seldom appears until two or three weeks after birth, when the infant shows evidences of a rhinitis, and gradually develops a snuffling and discharge from the nose. The victim suffers from malnutrition, looks aged and wizened, and frequently secondary eruptions appear, especially bullæ and vesicles on the palms and soles of the feet. Often destructive infiltrations occur in the septum nasi and other nasal structures, and in the floor of the nose, and the peculiar flat, distorted, sunken nose, with the projecting natiform frontal bosses, form the deformed but unmistakable "facies syphiliticæ."

Fortunately, seventy-five per cent. of these infants die before the second year; but those who live show remarkable changes in the form of the permanent central incisors. These are pegged, or notched, or irregularly developed, and are familiar to you as Hutchinson's teeth.

The subject of hereditary syphilis, you are to remember, is liable to develop any or all of the contagious secondary lesions, including mucous patches, and later the tertiary lesions.

It is contended by Colles that a child may inherit syphilis from the father, the mother remaining immune, immunized by the leucomaines and toxins produced in the infected child entering the mother's blood.

Profeta has shown that a child may be born free of or immune to syphilis, both parents being syphilitics prior to the tertiary stage.

It is contended that tertiary syphilis is not transmissible to the progeny.

These are very important matters in the practical consideration of the subject to the medical adviser and to the bench and bar.

I think I have shown that the dentist must, for his protection, be able to recognize syphilis when he sees it; also, that he must exercise extraordinary care to prevent infection; also, that his instruments are easily contaminated, and must be made surgically clean, to protect the non-syphilitic patient.

